# miR-34a is a common link in both HIV- and antiretroviral therapy-induced vascular aging

**DOI:** 10.18632/aging.101118

**Published:** 2016-11-28

**Authors:** Jiaxin Zhan, Shanshan Qin, Lili Lu, Xiamin Hu, Jun Zhou, Yeying Sun, Jian Yang, Ying Liu, Zunzhe Wang, Ning Tan, Jiyan Chen, Chunxiang Zhang

**Affiliations:** ^1^ Guangdong Key Laboratory of Coronary artery disease, Guangdong Cardiovascular Institute and Guangdong General Hospital, Guangzhou, 510100, China; ^2^ School of Medicine, Wuhan University of Science and Technology, Wuhan, 430081, China; ^3^ Department of Biomedical Engineering, School of Medicine, The University of Alabama at Birmingham, Birmingham, AL 35233, USA

**Keywords:** miR-34a, HIV, antiretroviral therapy, senescence, endothelial cells, vascular aging

## Abstract

Both HIV and antiretroviral therapy could induce vascular aging with unclear mechanisms. In this study, via microarray analysis, we identified, for the first time, that miR-34a expression was significantly increased in both HIV-infected, and antiretroviral agents-treated vessels and vascular endothelial cells (ECs) from these vessels. In cultured ECs, miR-34a expression was significantly increased by HIV-Tat protein and by the antiretroviral agents, lopinavir/ritonavir. Both HIV-Tat protein and antiretroviral agents could induce EC senescence, which was inhibited by miR-34a inhibition. In contrast, EC senescence was exacerbated by miR-34a overexpression. In addition, the vascular ECs isolated from miR-34a knockout mice were resistant to HIV and antiretroviral agents-mediated senescence. In vivo, miR-34a expression in mouse vascular walls and their ECs was increased by antiretroviral therapy and by HIV-1 Tat transgenic approach. miR-34a inhibition could effectively inhibit both HIV-Tat protein and antiretroviral therapy-induced vascular aging in mice. The increased miR-34a was induced via p53, whereas Sirt1 was a downstream target gene of miR-34a in both HIV-Tat protein and antiretroviral agents-treated ECs and vessels. The study has demonstrated that miR-34a is a common link in both HIV and antiretroviral therapy-mediated vascular aging.

## INTRODUCTION

Highly active antiretroviral therapy (HAART) has greatly reduced the risk of early death from opportunistic infections and extended the lifespan of people infected with immunodeficiency virus (HIV). Accordingly, cardiovascular complications in the HIV-infected population emerge due to the increased survival [1]. Indeed, both HIV and antiretroviral therapy could exacerbate vascular aging and its related cardio-vascular diseases such as atherosclerosis, coronary artery disease and stroke [2-4]. Vascular aging has thus become a matter of particular concern and a major cause of HIV-related death [5-8]. Despite intensive clinical and laboratory studies in the association among HIV infection, antiretroviral therapy and vascular aging, the molecular mechanisms of this significant clinical problem are largely unknown.

MicroRNAs (miRNAs) are a class of endogenous, small, non-coding RNAs that inhibit the expression of the protein coding genes via degradation or translational inhibition of their target messenger RNAs (mRNAs) [9]. miRNAs are highly expressed in the vascular system. The studies from our group and others have demonstrated that miRNAs may play important roles in vascular biology, vascular aging and vascular disease [10, 11]. Among the numerous discovered miRNAs, miR-34a has shown promise as a biomarker for organ aging, which also correlates with the impaired functions of vascular endothelial cells (ECs) from patients with vascular disease [12-16]. To date, the biological roles of miRNAs in both HIV and antiretroviral therapy-mediated vascular aging have not been explored.

By crossing-analysis of miRNA profiles of human and mouse arteries, and vascular ECs, with HIV-infection or antiretroviral therapy, we identified that miR-34a expression is significantly increased in both HIV-infected, and antiretroviral agents, ritonavir and lopinavir-treated vessels and cells. The Aim of this study is to determine the roles of miR-34a in both HIV and antiretroviral therapy-mediated vascular EC senescence and vascular aging.

## RESULTS

### miR-34a expression is significantly increased in both HIV-infected, and antiretroviral agents, ritonavir and lopinavir-treated human and mouse vessels and vascular ECs both in vitro and in vivo

**Figure 1:**
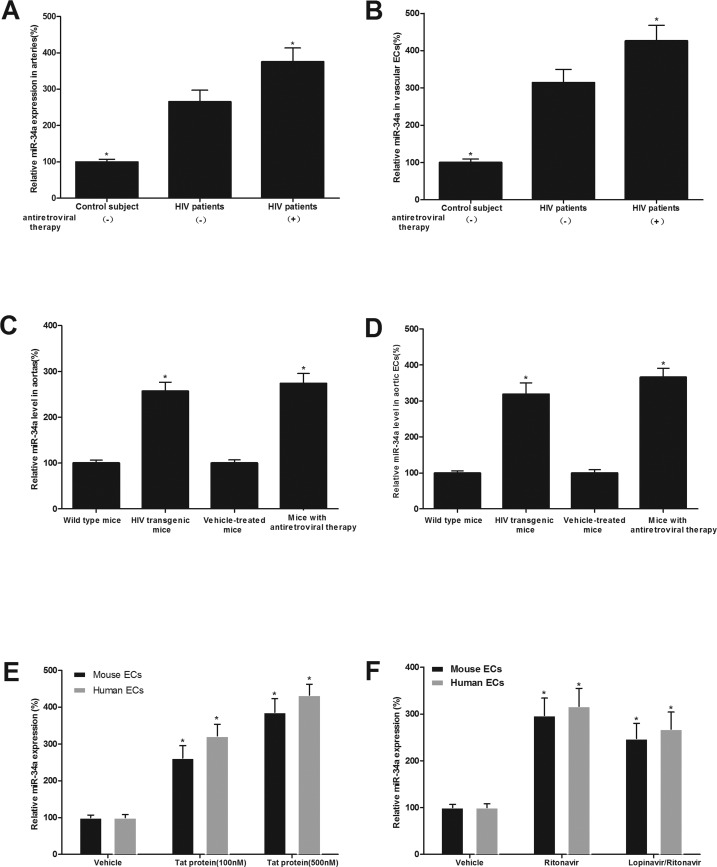
miR-34a expression is significantly increased in both HIV-infected, and antiretroviral agents, ritonavir and lopinavir-treated human and mouse vessels and vascular ECs both in vitro and in vivo miR-34a expression in arterial vessels (**A**) and in ECs isolated from these vessels (**B**) in HIV-infected patients with and without antiretroviral therapy (lopinavir/ritonavir, 800/200mg daily dose) and in their controls. miR-34a expression arterial vessels (**C**) and in ECs isolated from these vessels (**D**) in HIV-1 Tat transgenic mice and in mice with antiretroviral therapy (lopinavir/ritonavir, 125/31.25 mg/kg daily dose), as well as their controls. The effects of Tat1-101 (**E**), ritonavir or lopinavir plus ritonavir (lopinavir/ritonavir) (**F**) on the expression of miR-34a in human and mouse ECs. Note: n=6-9; *p<0.05 compared with the groups from HIV patients without antiretroviral therapy in (**A**) and (**B**), with the groups from wild-type mice or vehicle treated mice in (**C**) and (**D**), and with vehicle-treated groups in (**E**) and (**F**).

As shown in Fig.1A and 1B, miR-34a expression was significantly increased in arterial vessels and their isolated ECs from HIV-infected patients with and without antiretroviral therapy (lopinavir/ritonavir, 800/200mg daily dose). miR-34a expression was also increased in arterial vessels and their isolated ECs from HIV-1 Tat transgenic mice and from mice with antiretroviral therapy (lopinavir/ritonavir, 125/31.25 mg/kg daily via gavage for 4 weeks) (Fig. 1C and 1D).

The effect of HIV-Tat protein and antiretroviral agents on the expression of miR-34a was further verified in human and mouse aortic ECs. The cultured human and mouse ECs were treated with vehicle, recombinant Tat1-101 (100 nM), Tat1-101 (500 nM), ritonavir (7.5 μmol/L), or lopinavir (10 μmol/L) plus ritonavir (2 μmol/L), which are at clinically relevant concentrations, for 24 h. Then, the expression of miR-34a in ECs was determined by qRT-PCR. As shown in Fig. 1E and 1F, miR-34a in ECs was strongly increased by HIV-Tat protein and by antiretroviral agents.

### miR-34a has a strong promoting effect on senescence of cultured ECs

**Figure 2:**
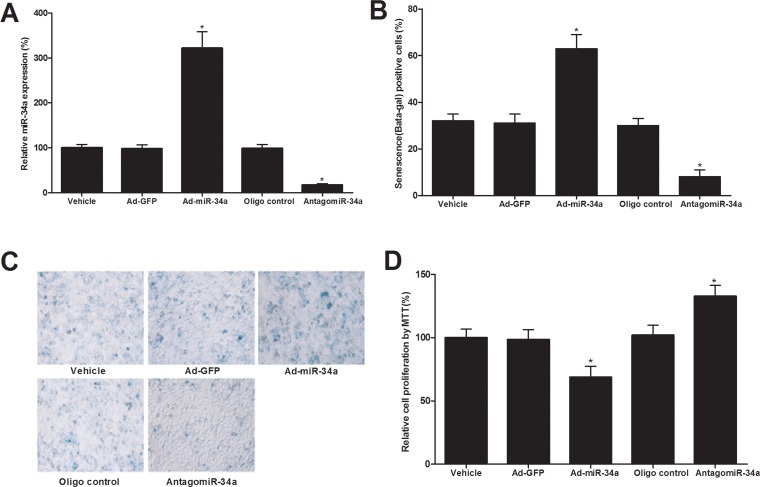
The effect of miR-34a on the senescence of cultured ECs (**A**) The expression of miR-34a in ECs was successfully increased by Ad-miR-34a (30 MOI) and decreased by AntagomiR-34a (30 nM). (**B**) The senescence of ECs as shown by senescence-associated beta-gal staining was significantly inhibited by miR-34a inhibition. In contrast, cell senescence was much exacerbated by miR-34a overexpression. (**C**) Representative images from beta-gal staining of ECs. (**D**) Cell proliferation was increased by AntagomiR-34a, but decreased by Ad-miR-34a. Note: n=6; *p<0.05 compared with control groups.

To determine the potential effect of miR-34a on the senescence of ECs, cultured human and mouse ECs were treated with vehicle, adenovirus control (Ad-GFP, 30 MOI), adenovirus expressing miR-34a (Ad-miR-34a, 30 MOI), AntagomiR-34a control (Oligo control, 30 nM), or miR-34a inhibitor AntagomiR-34a (30 nM). Seven days later, cell senescence induced by H_2_O_2_ (100 μM) was determined by senescence-associated beta-gal staining. In addition, cell proliferation was determined by MTT array as described [15]. As shown in Fig. 2A, the expression of miR-34a in ECs was successfully increased by Ad-miR-34a (30 MOI) and decreased by AntagomiR-34a (30 nM). The senescence of ECs was significantly inhibited by miR-34a inhibition. In contrast, cell senescence was much exacerbated by miR-34a overexpression (Fig. 2B). Representative images from beta-gal staining of ECs (cells with senescence) were shown in Fig. 2C. Accordingly, cell proliferation was increased by AntagomiR-34a, but decreased by Ad-miR-34a, which is consistent with the status of cell senescence (Fig. 2D).

### Both antiretroviral agents and HIV-Tat protein induce senescence of ECs, which could be inhibited by miR-34a knockdown or miR-34a knockout

**Figure 3:**
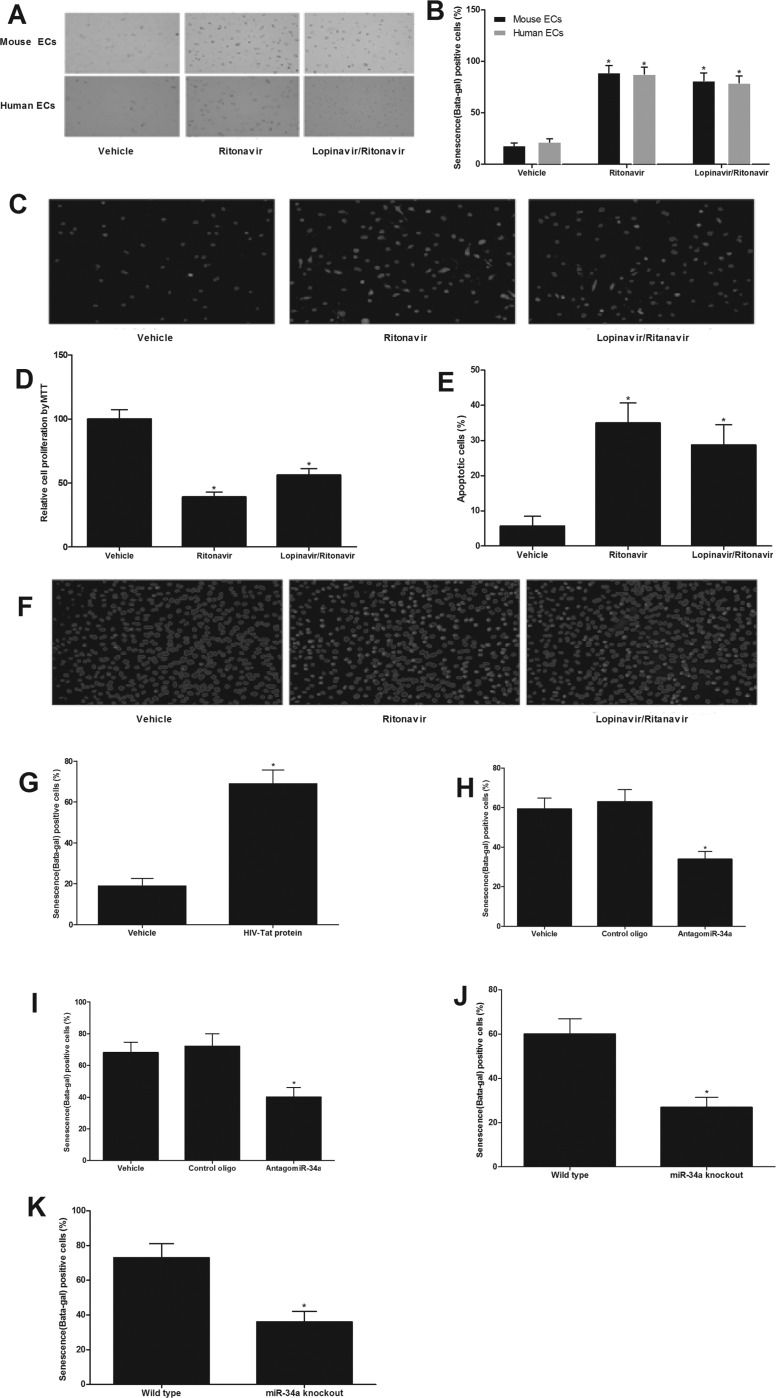
Both antiretroviral agents and HIV-Tat protein induce senescence of ECs, which could be inhibited by miR-34a knockdown or miR-34a knockout Antiretroviral agents ritonavir (7.5 μmol/L), or lopinavir (10 μmol/L) plus ritonavir (2 μmol/L) (lopinavir/ritonavir) could induce EC senescence as shown by the increased beta-gal staining (**A** and **B**), increased ROS production (**C**), decreased EC proliferation (**D**) and increased apoptosis induced by H2O2 (100 μM) (**E** and **F**). EC senescence was enhanced by HIV-Tat protein (100 nM) as shown by the increased beta-gal staining (**G**). Both antiretroviral agents (**H**) and HIV-Tat protein (**I**) induced senescence of ECs could be inhibited by miR-34a knockdown AntagomiR-34a (30 nM). The ECs from miR-34a knockout mice were resistant to antiretroviral agents (**J**) and HIV-Tat protein (**K**) induced senescence of ECs. Note: n=6; *p<0.05 compared with control groups.

In this experiment, cultured ECs were treated with vehicle, ritonavir (7.5 μmol/L), or lopinavir (10 μmol/L) plus ritonavir (2 μmol/L) (lopinavir/ritonavir) for 4 weeks. Then, the EC senescence was evaluated by beta-gal staining, proliferation, apoptosis, and ROS levels. As shown in Fig. 3A-3F, EC senescence was enhanced by antiretroviral agents. To set up the cell model of HIV-Tat protein-induced cell senescence, the cultured aortic ECs were treated with Tat1-101 (100 nM) or vehicle for 4 weeks. Then, the EC senescence was evaluated by beta-gal staining. As shown in Fig. 3G, EC senescence was enhanced by HIV-Tat protein. Interestingly, both antiretroviral agents and HIV-Tat protein induced senescence of ECs could be inhibited by miR-34a knockdown via AntagomiR-34a (30 nM) or miR-34a knockout by using the aortic ECs from miR-34a knockout mice (Fig. 3H-3K).

### Both antiretroviral agents-treated mice and HIV-Tat transgenic mice have the enhanced vascular aging, which could be inhibited by miR-34a knockdown in vivo

**Figure 4:**
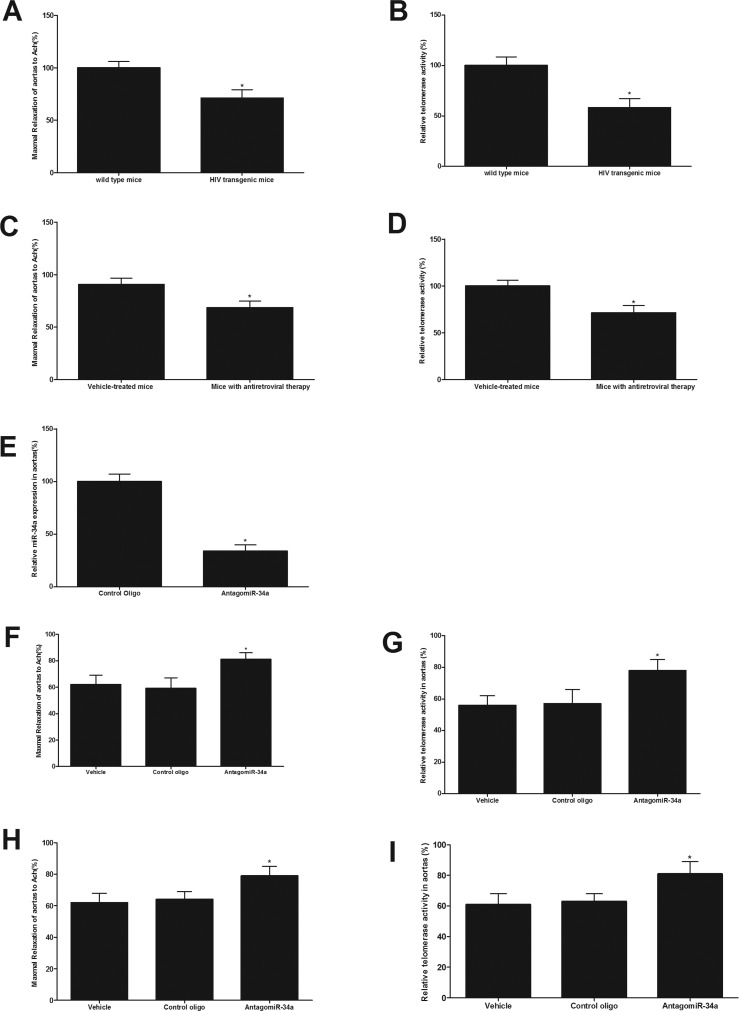
Both antiretroviral agents and HIV-Tat transgene have the enhanced vascular aging, which could be inhibited by miR-34a knockdown in mice in vivo HIV-1 transgenic mice have enhanced vascular aging as shown by the impaired endothelial function (**A**) and the reduced telomerase activity (**B**). Mice with antiretroviral therapy have the enhanced vascular aging as shown by the impaired endothelial function (**C**) and the reduced telomerase activity (**D**). The expression of miR-34a in mouse arteries was successfully down-regulated by AntagomiR-34a (40 mg/kg, iv per week, via tail vein) in vivo (**E**). Compared with that from vehicle treated mice, mice with antiretroviral therapy had the enhanced vascular aging as shown by the impaired endothelial function **(F**) and the decreased telomerase activity (**G**), which was inhibited via miR-34a knockdown. Compared with that from vehicle treated mice, vascular aging was significantly inhibited via miR-34a knockdown in HIV-1 Tat transgenic mice (**H** and **I**). Note: n=6-8; *p<0.05 compared with control groups.

HIV-1 transgenic mice had the enhanced vascular aging as shown by the impaired endothelial function (Fig. 4A) and the reduced telomerase activity (Fig. 4B). In addition, mice with antiretroviral therapy (lopinavir/ritonavir, 125/31.25 mg/kg daily dose, via gavage, for 8 weeks) also had the enhanced vascular aging as shown by the impaired endothelial function (Fig. 4C) and the reduced telomerase activity (Fig. 4D). The expression of miR-34a in mouse arteries could be successfully down-regulated by AntagomiR-34a (40 mg/kg, iv per week) in vivo (Fig. 4E). As shown in Fig. 4F and 4G, compared with that from vehicle treated mice, antiretroviral therapy-induced vascular aging could be inhibited in part by miR-34a knockdown (40 mg/kg, iv per week for 3 weeks). To determine the role of miR-34a in HIV-1 Tat-induced vascular aging, HIV-1 Tat transgenic mice were used. The animals were treated with AntagomiR-34a (40 mg/kg, iv per week for 3 weeks), vehicle or control oligo (40 mg/kg, iv per week for 3 weeks). As shown in Fig. 4H and 4I, compared with that from vehicle treated mice, vascular aging was inhibited via miR-34a knockdown in HIV-1 Tat transgenic mice.

### p53 is an upstream signaling molecule that is responsible for the increased miR-34a in both HIV-Tat protein and antiretroviral agents-treated ECs and vessels

**Figure 5:**
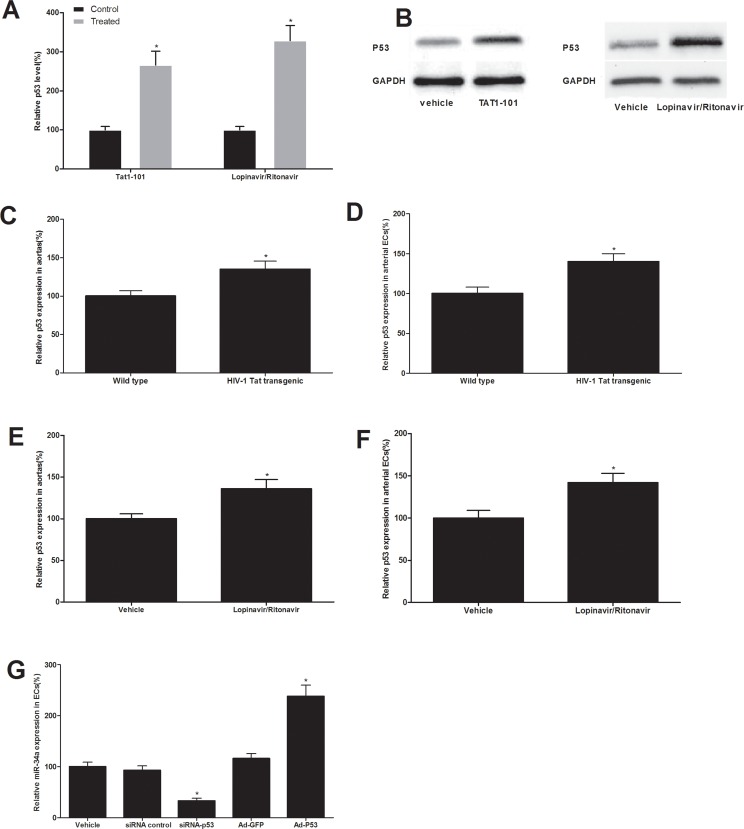
p53 is an upstream signaling molecule that is responsible for the up-regulation of miR-34a in both HIV-Tat protein and antiretroviral agents-treated ECs and vessels The expression of p53 was increased in ECs treated with Tat1-101 or lopinavir/ritonavir (**A** and **B**). The expression of p53 was also increased in vascular walls and their ECs from HIV-1 Tat transgenic mice (**C** and **D**) and from mice with antiretroviral therapy in vivo (**E** and **F**). The expression of miR-34a was decreased by p53 knockdown, but was increased by p53 over expression (**G**). Note: n=6; *p<0.05 compared with control groups.

P53 is a known critical regulator for miR-34a synthesis [16]. To determine whether or not the p53 plays a role in the up-regulation of miR-34a in both HIV-Tat protein and antiretroviral agents-treated ECs and vessels, we first determined the expression change of p53 in Tat1-101-treated ECs and in ECs treated with lopinavir/ritonavir. The result revealed that p53 was increased in Tat1-101-treated ECs and in ECs treated with antiretroviral agents (Fig. 5A and 5B). In addition, the expression of p53 was also increased in vascular walls and their ECs from HIV-1 Tat transgenic mice (Fig. 5C and 5D) and from mice with antiretroviral therapy in vivo (Fig. 5E and 5F). To provide the direct evidence that p53 is indeed an upstream regulator for miR-34a expression in these ECs, we purchased the siRNA for p53 knockdown and created the Ad-p53. As shown in our recent report [17], the p53 was successfully knocked down by its siRNA (siRNA-P53) and was significantly up-regulated by Ad-p53 in vascular cells. The cultured ECs were treated with vehicle, siRNA control (50 nM), siRNA-P53 (50 nM), adenovirus control Ad-GFP (30 MOI) or Ad-P53. At 48 hours after treatment, the expression of miR-34a in cells was determined by qRT-PCR. As shown in Fig. 5G, the expression of miR-34a was decreased by p53 knockdown, but was increased by p53 over expression. The results suggested that p53 is indeed an important upstream signaling mechanism responsible for the increased miR-34a in both HIV-Tat protein and anti-retroviral agents-treated ECs and vessels.

### Sirt1 is a direct target gene of miR-34a that is related to HIV-Tat protein and antiretroviral agents-induced vascular aging

**Figure 6:**
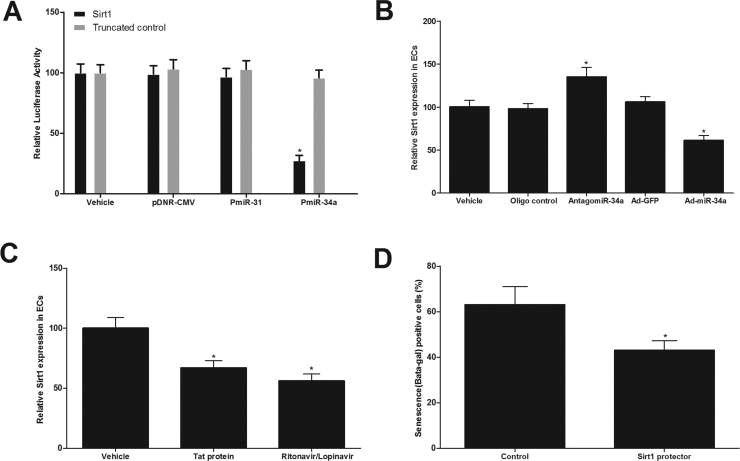
Sirt1 is a direct target gene of miR-34a that is related to HIV-Tat protein and antiretroviral agents-induced vascular aging (**A**) pmiR-miR-34a, but not pmiR-31 or pDNR-CMV, inhibited luciferase activity. (**B**) the expression of Sirt1, was increased by miR-34a knockdown via antagomiR-34a (30 nM), but was decreased by miR-34a over expression via Ad-miR-34a (30 MOI). (**C**) The expression of Sirt1 in ECs was decreased by HIV-Tat protein or antiretroviral agents. (**D**) miR-34a-mediated senescence of cultured ECs was partially inhibited by the protector of Sirt1(30 nM). Note: n=6; *p<0.05 compared with control groups.

Computational analysis via online software such as TargetScan 5.1 predicts that Sirt1 has the miR-34a binding site in their 3′-untranslated region (3′-UTR), whereas Sirt1 is critical gene for EC senescence and vascular aging [18]. To test whether or not the Sirt1 is a direct target gene of miR-34a, we first performed a luciferase assay. The result demonstrated that pmiR-miR-34a, but not pmiR-31(an un-related control miRNA) or pDNR-CMV, inhibited luciferase activity (Fig. 6A). In the mutated control group (truncated control), the inhibitory effect of pmiR-miR-34a disappeared. The results suggested that miR-34a could directly bind to and inhibit the expression of Sirt1. Secondly, the effect of miR-34a on the expression of Sirt1 in cultured ECs was determined. As shown in Fig. 6B, the expression of Sirt1, was increased by miR-34a knockdown via antagomiR-34a (30 nM), but was decreased by miR-34a over expression via Ad-miR-34a (30 MOI). In addition, the expression of Sirt1 in ECs was decreased by HIV-Tat protein or antiretroviral agents (Fig. 6C). Finally, to determine whether the Sirt1 is a functional target gene related to HIV-Tat protein and antiretroviral agents-induced vascular aging, the cultured ECs were pre-treated with target protector control (30 nM), or Sirt1 protector (30 nM), which could block the binding of p34 with Sirt1. Then, the cells were treated with Ad-miR-34a (30 MOI) for 2 weeks. After that, the cell senescence was determined. As shown in Fig. 6D, miR-34a-mediated senescence of cultured ECs was partially inhibited by its protector. The results suggested that Sirt1 was indeed involved in miR-34a-mediated effect on vascular aging.

## DISCUSSION

Both HIV itself and antiretroviral therapy could exacerbate vascular aging and its related vascular diseases. Despite intensive clinical and laboratory studies in the association among HIV infection, antiretroviral therapy and vascular aging, the molecular mechanisms of this significant clinical problem are largely unknown. This has highlighted the importance and urgency of studying the novel mechanisms of HIV and antiretroviral therapy-induced vascular aging and exploring new therapeutic options to improve our current HIV-therapy. We hypothesize that HIV and antiretroviral therapy might have a common mechanism at molecular level, in which miRNAs might be potential candidates. However, up to now, the biological roles of miRNAs in both HIV and antiretroviral therapy-mediated vascular aging have not been explored.

By crossing-analysis of miRNA profiles of human and mouse arteries, and vascular ECs, with HIV-infection or antiretroviral therapy, we have identified miR-34a expression is significantly increased in both HIV-infected, and antiretroviral agents, ritonavir and lopinavir-treated vessels and cells. We thus hypothesize that miR-34a may play a shared role in both HIV and antiretroviral therapy-mediated vascular aging.

MiR-34a is a critical miRNA related to vascular EC senescence and vascular aging. To test the potential roles of miR-34a in both HIV and antiretroviral therapy-mediated vascular aging, we first determined the effect of miR-34a on senescence in cultured human and mouse ECs by both gain-of-function and loss-of-function approaches. The results have demonstrated that miR-34a has a strong promoting effect on EC senescence. To provide a direct link between miR-34a and EC senescence induced by HIV and antiretroviral therapy, the cultured mouse and human ECs were treated with either HIV-Tat protein Tat1-101 or antiretroviral agents, ritonavir and lopinavir. The results have revealed that both Tat1-101 and ritonavir/lopinavir are able to induce EC senescence, and in the meantime, increase the expression of miR-34a in these cells. Interestingly, both HIV-Tat protein and antiretroviral agent-induced EC senescence could be effectively inhibited by knockdown of miR-34a. The effect of miR-34a on EC senescence is further verified by using miR-34a knockout approach, in which the ECs without miR-34a are resistant to both HIV-Tat protein and antiretroviral agent-induced senescence.

The expression of miR-34a in vascular wall and their ECs from HIV patients, antiretroviral agent-treated human subjects, antiretroviral agent-treated mice and HIV-Tat transgenic mice is significantly increased. Also, these vessels have enhanced vascular aging. To provide the potential direct link of miR-34a and vascular aging in vivo, the expression of miR-34a was knocked down by its inhibitor in mice. Remarkably, HIV and antiretroviral agent–induced vascular aging is able to be partially inhibited.

Although the results from our study have shown a clear shared role of miR-34a in both HIV and antiretroviral agent–induced vascular aging, the vascular aging cannot be completely blocked via miR-34a knockdown or knockout. It suggests that other molecular mechanisms including other miRNAs should be determined in future studies.

One critical question is that why is miR-34a increased in both HIV infected and antiretroviral agent-treated vessels? Recent studies have suggested that p53 could be an important positive regulator for the expression of miR-34a [16]. Interestingly, our data has demonstrated that both HIV-1 Tat and antiretroviral therapy (lopinavir/ritonavir) could increase the expression of p53 in cultured ECs in vitro and in vascular walls in vivo. To provide the direct evidence that p53 is a critical upstream regular for the expression of miR-34a, the levels of p53 was modulated via its siRNA and Ad-p53. We have found that the expression was decreased by p53 knockdown, but was increased by p53 over expression. The results suggest that p53 is indeed an important upstream signaling molecule responsible for the increased miR-34a in both HIV-Tat protein and antiretroviral agents-treated ECs and vessels. However, which step of miR-34a biogenesis is affected by p53 is still unclear.

It is well known that a miRNA achieves its biological functions via its multiple target genes. Based on the biological function of miR-34a and the computational analysis, we have found that Sirt1 has a miR-34a binding site in its 3′-untranslated region (3′-UTR). Thus, Sirt1 could be a potential direct target gene of miR-34a in ECs. The link between mir-34a and Sirt1 was also reported by the reports from other groups [21, 22]. The ability of that miR-34a can bind to the fragments of the 3′-UTR of Sirt1 and inhibit its expression is confirmed by luciferase assay. In addition, the expression of Sirt1 is decreased by miR-34a in ECs. Finally, the functional involvement of Sirt1 in miR-34a-mediated effect on EC senescence is verified by the special target protector for it. These results suggested that Sirt1 is the functional direct target of miR-34a in ECs.

In Summary, p53 is activated by HIV infection and antiretroviral therapy. The up-regulated p53 is able to increase the expression of miR-34a in vascular walls and their ECs. The increased miR-34a could induce the EC senescence and vascular aging in patients and animals with HIV infection and antiretroviral therapy via down-regulation of its direct target genes such as Sirt1. It is possible that new modalities may be developed to prevent and treat the HIV-and antiretroviral therapy induced vascular aging and vascular disease by targeting miRNAs such as miR-34a.

## METHODS

### Animals and animal models

Wild-type male C57BL/6 mice (8–10 weeks, 25–28 g), HIV-1 Tat transgenic mice and miR-34a knockout mice were from Jackson Laboratory. To induce vascular aging in normal wild type mice, one group of animals were provided the antiretroviral therapy (lopinavir/ritonavir at 125/31.25 mg/kg, Santa Cruz bio-technology) via oral gavage for 12 weeks starting at 12-week-old. The dose was based on human dosing guidelines for daily oral lopinavir/ritonavir in adult HIV patients (800/200 total mg or 10/2.5 mg/kg), and on body surface area (BSA) normalization factors of mice, which translate 10 mg/kg in humans to approximately 125 mg/kg in mice. The animal protocol was approved by the Institutional Animal Care and Use Committee and was consistent with the Guide for the Care and Use of Laboratory Animals (NIH publication 85–23, revised 1985).

### Cell isolation and culture

Endothelial cells (ECs) were isolated from mouse aortas and human arteries from patients with arteriosclerosis obliterans (ASO) with and without HIV under sterile conditions by established techniques as described in our recent report [23]. All the ECs were verified by the expression of endothelium-specific markers such as Ve-cadherin and CD31 and cultured with Cell Biologics’ complete growth medium for ECs. All the cell isolated from human and mice in this study were described in results section. In addition, some normal mouse and human arterial ECs were purchased from American Type Culture Collection (ATCC) and were used in some experiments in vitro. The cells were cultured with medium provided by ATCC.

### RNA isolation and qRT‐PCR assay

RNAs from cultured cells and arteries, mouse carotid arteries and aortas, and human arteries were isolated with TRIzol (Life Technologies, Carlsbad, CA). For miRNA, cDNA was generated from 100 ng of total RNA using TaqMan MiRNA Reverse Transcription and TaqMan MiRNA assays (Life Technologies, Carlsbad, CA) [24-26]. For other RNAs, cDNA was generated from 200 ng of total RNA using High-Capacity RNA-to-cDNA Kit (Life Technologies, Carlsbad, CA). qRT-PCR for both miRNA and mRNA were performed on cDNAs using TaqMan Fast Universal PCR Master Mix (2X), no AmpErase UNG (Life Technologies, Carlsbad, CA), according to the manufacturer's instructions. Amplification and detection of specific products were performed with a Life Technologies 480 ViiA 7 Detection System (Life Technologies, Carlsbad, CA). As an internal control, U6 was used for miRNA template normalization and GADPH was used for other template normalizations. Fluorescent signals were normalized to an internal reference, and the threshold cycle (Ct) was set within the exponential phase of the PCR. Relative gene expression was calculated by comparing cycle times for each target PCR. The target PCR Ct values were normalized by subtracting the U6 or GADPH Ct value, which provided the ΔCt value. Relative expression between treatments was then calculated using the following equation: relative gene expression=2^−(ΔCt sample−ΔCt control)^.

### Construction of the adenoviruses

The adenoviruses expressing miR-34a, p53 and control viruses expressing GFP (Ad-GFP) were generated using the Adeno-X™ Expression Systems 2 kit (Clontech, CA) according to the manufacturer's protocols as described previously [17, 19]. The resulting adeno-viruses were further amplified by infection of HEK293A cells and purified by cesium chloride gradient ultracentrifugation. The Ad-miR-34a, Ad-p53 and Ad-GFP were titrated using a standard plaque assay.

### Oligonucleotide transfection and gene modulation in cultured ECs

Oligonucleotide transfection was performed as described in our previous studies [24-26]. Briefly, cells were transfected using a transfection reagent (Qiagen, Valencia, CA) 24 hours after seeding into the wells. Transfection complexes were prepared according to the manufacturer's instructions. The expression of miR-34a was downregulated by its inhibitor (AntagomiR-34a) (30 nM), and was upregulated via Ad-miR-34a (30 MOI) (Vector BioLabs, Malvern, PA). Gene knock-down for other genes was performed using their siRNA (50 nmol/L; Invitrogen, Carlsbad, CA). The transfection medium was replaced 4 hours posttransfection by the regular culture medium. Vehicle, scramble and Ad-GFP controls (30 MOI) were applied.

### Senescence-associated β-galactosidase staining assay

Cell senescence in cultured cell in vitro and in vessel sections was examined with the senescence-associated β-galactosidase (β-gal) staining Kit (Cell Signaling, Danvers, MA), according to the manufacturer's instructions [13]. Briefly, the cells and freeze vessel sections were washed twice with PBS and fixed for 15 min in room temperature, then washed again to remove the fixing solution, incubated in the SA-β-gal staining solution for overnight at 37°C and viewed under the Nikon microscope. The β-gal positive (blue color) cells were counted and the β-gal staining densities were calculated by computerized image analysis, using ImageJ™ software (NIH, MD).

### The measurement of intracellular reactive oxygen species (ROS)

The generation of intracellular ROS in cardiomyocytes was measured using the Image-iT ™ LIVE Green Reactive Oxygen Species Detection Kit (Molecular Probes, Eugene, OR) [27]. The assay was based on 5-(and-6)-carboxy-2′,7′-dichlorodihydrofluoresceindia-cetate (carboxy-H2DCFDA), a reliable fluorogenic marker for ROS in live cells. The cell at culture slides were washed twice with phosphate-buffered saline and stained with 10 μM carboxy-H2DCFDA at 37°C for 30 min. The fluorescence images were obtained using microscopes (Eclipse Ti-U, Nikon).

### Cell proliferation and apoptosis

EC proliferation was determined by 3-[4,5-dimethylthiazol-2-yl]-2,5-diphenyltetrazolium bromide; thiazolyl blue (MTT) assay [17], cell counting, and BrdU assay. EC apoptosis in cultured cells was induced by treatment with H_2_O_2_ (100 μM) for 24 hours. Apoptosis was measured by Terminal deoxynucleotidyl transferase dUTP nick end labeling (TUNEL) analysis as described [17]. Apoptotic cells were quantified by counting the percentage of TUNEL-positive cells against total nucleated cells stained by DAPI.

### Telomerase activity

Telomerase activity in vessel tissues was quantified using the revised real-time PCR Telomerase Repeat Amplification Protocol (TRAP) as described [28]. Protein extracts (0.1μg) of mouse hearts or 10,00 cardiomyocytes were used. Real-time TRAP was done in a ViiA™ 7 Real-Time PCR System (Applied Biosystems, Waltham, MA). The reaction mixture was first incubated at 30°C for 30 minutes to allow the telomerase in the protein extracts to elongate the TS primer by adding TTAGGG repeat sequences. The PCR was then initiated at 95°C for 20 seconds to activate the modified Taq polymerase, followed by 40 cycles of 95°C for 1 second, 60°C for 20 seconds, and one cycle of 60°C for 15 seconds and 0.1°C/s to 95°C. SYBR Green bound to the new amplicons and generated fluorescent signals that were collected and analyzed. A standard titration curve was established via 0 to 10,000 cells/0.001 to 10ug tissue protein extracts to ensure linearity of the assay.

### miR-miR-34a inhibition in mice in vivo

miR-34a expression in vivo in mice was knocked down by the in vivo microRNA Inhibitor AntagomiR-34a. AntagomiR-34a (40 mg/kg) (Integrated DNA Technologies) was injected into C57 mice via tail vein.

### Endothelial function assessment in isolated mouse aortas

Isometric tension was measured in isolated mouse aortic ring segments as described [29, 30]. The vessels were cut into individual ring segments (2–3 mm in width) and suspended from a force-displacement transducer in a tissue bath. Ring segments were bathed in Krebs-Henseleit (K-H) solution. The vessels were contracted to 50–60% of their maximal capacity (50–60% of KCl response) with phenylephrine (3×10^−8^−10^−7^ M). When tension development reached a plateau, ACh (10^−9^−3×10^−6^ M) was added cumulatively to the bath to stimulate endothelium-dependent relaxation.

### Luciferase assay

The reporter plasmid, a firefly luciferase reporter construct psiCHECK-2 (Promega, WI) inserted with a fragment of the 3′-UTR of mouse *Sirt1* mRNA containing the putative miR-34a binding sequence. The construct with mutated fragment of the 3′-UTR of *Sirt1* mRNA without the putative miR-34a binding sequences was used as the mutated control. HEK 293 cells were transfected with the construct or the mutated control construct. Then, these HEK 293 cells were treated with vehicle, pDNR-CMV (an empty plasmid, 0.2 μg/ml), or pmiR-34a (a plasmid expressing miR-34a, 0.2 μg/ml). Cell extract was isolated to measure the luciferase expression on a scintillation counter by using a dual luciferase reporter system.

### Western blot analysis

Proteins isolated from endothelial cells and vessels were determined by Western blot analysis. Equal amounts of protein were subjected to SDS-PAGE. A standard Western blot analysis was conducted using their antibodies. GAPDH antibody (1:5000 dilution; Cell Signaling) was used as a loading control.

### Human samples

This study was approved by the research ethics committee and was performed conform the declaration of Helsinki. The human arterial vessels and their isolated ECs were isolated from HIV patients with arteriosclerosis obliterans (ASO) during surgery. The normal lower limb artery samples were acquired from donors without HIV infection were used as controls. All data were de-identified before being provided to the investigators with the informed consent of all subjects.

### Statistical analysis

All data are expressed as mean ± SEM (standard error of the mean). All the experiments were repeated independently at least three times. For relative gene expression, the mean value of control group is defined as 100%. SPSS was used to perform the statistical analysis. ANOVA repeated measures were used to assess changes within a group, and one-way ANOVA within groups were used to assess the significance of any change between groups. Comparisons between two groups were performed using the independent samples t-test. Statistical significance was accepted at *P* < 0.05.
